# Distribution of intraocular pressure in a Swedish population

**DOI:** 10.48101/ujms.v127.8829

**Published:** 2022-10-03

**Authors:** Maria Häkkinen, Curt Ekström

**Affiliations:** Department of Surgical Sciences, Ophthalmology, Uppsala University, Uppsala, Sweden

**Keywords:** Diabetes, epidemiology, intraocular pressure, open-angle glaucoma, population survey, pseudoexfoliation, repeated applanation tonometry, risk factor

## Abstract

**Background:**

Increased intraocular pressure (IOP) and pseudoexfoliation (PEX) are major risk factors for open-angle glaucoma (OAG), an age-related neurodegenerative disease of significant importance for public health. There are few studies on the distribution of IOP in populations where PEX is a common finding.

**Methods:**

The distribution of IOP was studied in 733 subjects 65–74 years of age, examined in a population survey in the rural district of Tierp, Sweden, 1984–86. The difference between the right and left eye and the effect of which eye was measured first were examined. Odds ratios, adjusted for age and sex, according to Mantel-Haenszel (OR_MH_), were calculated to estimate predictors of increased IOP, defined as a pressure ≥20 mm Hg in either eye. The pressure was measured with Goldmann applanation tonometry. Automated perimetry was used to identify OAG.

**Results:**

The distribution of IOP was close to that of other European-derived populations. The pressure in the first measured eye was higher than in the second measured eye. Increased IOP was related to OAG and PEX, OR_MH_ 8.97 (95% confidence interval [CI] 3.84–20.9) and 2.40 (95% CI 1.53–3.76), respectively. An IOP ≥20 mm Hg increased the risk of having been diagnosed with diabetes (OR_MH_ 1.83; 95% CI 1.08–3.09).

**Conclusion:**

In this study of subjects 65–74-years-old in Sweden, the distribution of IOP was close to that of other European-derived populations. Although the difference was small, the pressure in the first measured eye was higher than in the second eye. Increased IOP was strongly related to untreated OAG and PEX.

## Introduction

Open-angle glaucoma (OAG) is an age-related neurodegenerative disease of significant importance for public health, characterised by progressive loss of optic nerve fibres with typical appearance of the optic nerve head and consistent visual field defects. Globally, glaucoma is the leading cause of irreversible blindness (1). In a Swedish study, increased intraocular pressure (IOP) and pseudoexfoliation (PEX) were proved to be important risk factors for the development of OAG (2). In PEX, a fibrillar material is produced and accumulated in the anterior segment of the eye, thus increasing the pressure by impairing the outflow of aqueous humour (3). Common sequence variants in the lysyl oxidase-like 1 gene, involved in elastic fibre formation, are closely related to PEX (4).

The distribution of intraocular pressure is well-known from numerous studies on different ethnicities. One of the earliest studies, conducted in Ferndale in Wales, reported a mean IOP of 16.6- and 15.9-mm Hg for women and men, respectively (5). Results from the studies in Framingham and Beaver Dam on subjects 65–74 years old are presented in Table 1 (6, 7). To the best of our knowledge, only two studies on the distribution of IOP, using applanation tonometry, have been done in Sweden, both from Dalby in the south (8, 9). The first of these studies reported a mean IOP in the right eye of 15.4 mm Hg for the age group 60–69 years and 15.9 for the age group 70–79 years. Pseudoexfoliation was an uncommon finding in the Dalby population (9).

**Table 1:** Percent distribution of intraocular pressure in the right eyes in individuals aged 65–74 years in the Framingham eye study and the Beaver Dam eye study by sex.

Study	IOP (mm Hg)							
	Sex	<13	13–15	16–18	19–21	22–24	≥25	Mean
Framingham^a^	Females	10.5	25.4	35.8	19.7	5.0	3.6	17.0^c^
	Males	13.4	25.3	34.3	17.4	4.7	4.9	16.7^c^
Beaver Dam^b^	Females	13.8	30.9	36.4	13.4	4.2	1.2	16.0
	Males	18.3	34.0	31.2	10.9	5.1	0.6	15.5

IOP: intraocular pressure.

a Ref. (6);

b Ref. (7); the age groups 65–69 years and 70–74 years are combined;

c The mean relates to both eyes.

A connection between PEX and increased IOP has been demonstrated in several population studies (10–12). However, there are few studies on the distribution of IOP in populations where PEX is a common finding, none of them from Sweden (13–15). The study in Oulu, in the north of Finland, reported a mean pressure of 16.2 mm Hg in the right eye and 15.7 in the left eye (13). A follow-up study in Skellefteå in northern Sweden, where PEX is common, found a mean IOP of 16.3 mm Hg in women and 15.3 in men at baseline, when the subjects were 66 years old (16).

The objectives of the present research were to examine the distribution of IOP in a Swedish population with a high exposure to PEX and to estimate predictors of increased pressure. The effect of which eye was measured first was also studied. The investigation took the form of a cross-sectional study on a defined population.

## Methods

### The Tierp Glaucoma Survey

In 1984–1986, a population survey was conducted in the rural district of Tierp, south central Sweden. Its target population comprised 2,429 residents, aged 65–74-years-old. A sample of about one-third of the target population was randomly selected. Of the eligible number of 838 individuals, 760 (91%) underwent a detailed eye examination, as described elsewhere (17). Briefly, an interview was first held, covering medical and family history. The pressure was taken with a Goldmann applanation tonometer mounted on a Haag–Streit slit lamp. In subjects 65–69-years old, whose date of birth was divided by the figure 2, the left eye was measured first, while in the rest of the sample, the right eye was measured first. As a rule, the pressure was taken with single tonometer readings. If the difference between the two eyes exceeded 2 mm Hg, a control measurement was done, as described by Bengtsson (8). In this case, the second reading was defined as the IOP for that person.

The visual fields were tested using the Competer 350 automated perimeter (Bara Elektronik AB, Lund, Sweden). After perimetry, the pupils were dilated, and the slit lamp biomicroscopy, including a binocular assessment of the optic discs and gonioscopy, was done. The presence of cataract was ascertained based on retroillumination using indirect ophthalmoscopy with lens opacities evident on biomicroscopy. Pseudoexfoliation was defined as the presence of characteristic white flakes on the lens capsule or on the pupillary border.

### The study population

**Figure 1:**
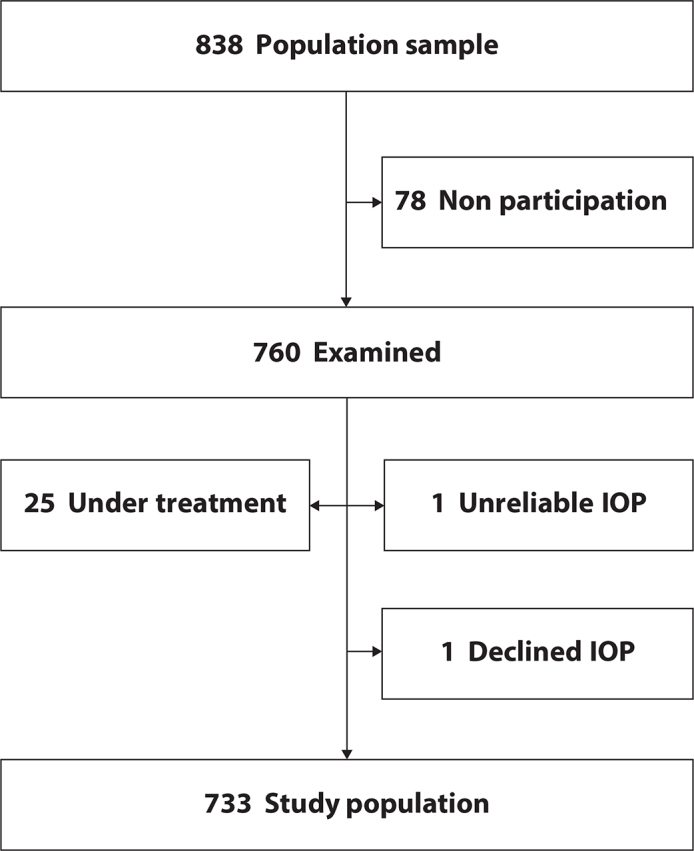
Flow chart showing how the study population of 733 individuals was derived. IOP: intraocular pressure.

Of the total number of 760 participants, 25 were treated for glaucoma. These subjects were excluded from the study, as was one subject with unreliable pressure readings of both eyes. One individual declined IOP measurement (Figure 1). The remaining 733 people, 381 women and 352 men, constituted the study population. The investigation was approved by the Human Subjects Committee at the Faculty of Medicine, Uppsala University, and adhered to the tenets of the Declaration of Helsinki. An informed consent was obtained from all participants. This report is in accordance with the original ethical approval.

### Classification of OAG

Consistent with the concept of Foster et al. (18), glaucoma with PEX was classified as OAG. To qualify for a diagnosis of OAG, a reproducible visual field defect was a prerequisite, consistent with glaucoma and not explicable on other grounds, as described elsewhere (17). Twenty-four subjects fulfilled a diagnosis of definite OAG. Pseudoexfoliation in either eye was present in 117 subjects (16.0%), of whom five were diagnosed with OAG.

## Assessment of systemic predictors

Information on treated systemic hypertension, ischaemic heart disease, and diabetes mellitus was obtained at the interview or from medical records. In the case of a discrepancy between the self-reported history and the medical record, data from the latter source were used in this report. The participants were asked if they were current smokers or past smokers and when they stopped smoking. Information on smoking was also acquired from medical records and family members.

### Statistical methods

A repeated measures ANOVA was done to explore the covariation in IOP between the right and left eye depending on which eye was measured first. Predictors of increased IOP, defined as a pressure ≥20 mm Hg in either eye, were estimated using 2 × 2 tables, with odds ratios adjusted for age and sex strata, according to the Mantel–Hansel’s method (OR_MH_). To simultaneously assess several variables affecting the risk for increased IOP, multiple logistic regression analyses were used, with an IOP ≥20 mm Hg as the dependent variable.

## Results

**Figure 2:**
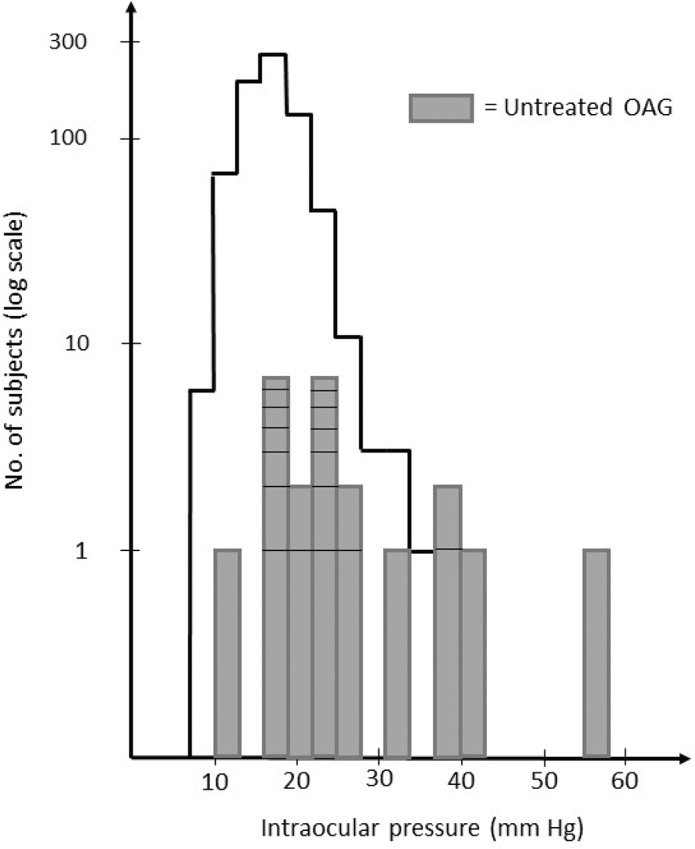
Distribution of the highest pressure in either eye in the study population of 733 participants in the Tierp Glaucoma Survey (3 mm Hg pressure intervals). Twenty-seven individuals were excluded. OAG: open-angle glaucoma.

The distribution of the highest pressure in either eye was slightly drawn-out to the right, as shown in Figure 2. Most of the OAG cases were found to the right. The median pressure was 17 mm Hg (interquartile range 15–19), and the mean pressure was 16.9 (95% CI 16.7–17.2). The percent distribution in the right eyes is presented in Table 2. There were small differences between individuals aged 65–70 years and 70–74 years, and between females and males. In subjects 65–69 years of age, the mean pressure was higher in the eye that was measured first (Table 3). Analysis of variance revealed a small but significant interaction between measuring the right eye first and the left eye second (*P* = 0.0025). The mean IOP in right eyes with PEX was 18.7 mm Hg, compared with 16.0 in eyes without PEX, with a clear overrepresentation of pressures above 21 mm Hg in eyes with PEX (Table 4).

**Table 2:** Percent distribution of intraocular pressure in right eyes in 731 participants in the Tierp Glaucoma Survey by age and sex.^a^

Age (Years)	IOP (mm Hg)								
	Sex	No.	<13	13–15	16–18	19–21	22–24	≥25	Mean
65–69	F	202	9.4	29.2	34.7	21.8	3.0	2.0	16.6
65–69	M	187	12.3	32.6	33.2	12.8	6.4	2.7	16.4
65–69	Total	389	10.8	30.8	33.9	17.5	4.6	2.3	16.5
70–74	F	178	17.4	26.4	37.6	9.0	5.6	3.9	16.3
70–74	M	164	16.5	37.8	27.4	12.8	4.3	1.2	15.7
70–74	Total	342	17.0	31.9	32.7	10.8	5.0	2.6	16.0
65–74	F	380	13.2	27.9	36.1	15.8	4.2	2.9	16.5
65–74	M	351	14.2	35.0	30.5	12.8	5.4	2.0	16.1
65–74	Total	731	13.7	31.3	33.4	14.4	4.8	2.5	16.3

IOP: intraocular pressure; F: females; M: males.

a Twenty-nine subjects are excluded; the right eye was removed in two subjects.

**Table 3:** Mean intraocular pressure in the right and left eye in 389 participants 65–69 years of age in the Tierp Glaucoma Survey by the first measured eye.^a^

Eye	Right eye measured first			Left eye measured first		
	No.	IOP	(95% CI)	No.	IOP	(95% CI)
Right eye	200	16.7	(16.1–17.3)	189	16.3	(15.8–16.8)
Left eye	200	16.2	(15.7–16.7)	189	16.5	16.0–17.0

IOP: intraocular pressure, mm Hg; CI: confidence interval.

a Fifteen subjects treated for glaucoma are excluded from the analyses.

**Table 4:** Percent distribution of intraocular pressure in right eyes in 731 participants in the Tierp Glaucoma Survey by the presence of pseudoexfoliation.^a^

PEX	IOP (mm Hg)							
	No.	<13	13–15	16–18	19–21	22–24	≥25	Mean
Yes	79	11.4	25.3	25.3	13.9	10.1	13.9	18.7
No	652	14.0	32.1	34.4	14.4	4.1	1.1	16.0

IOP: intraocular pressure; PEX: pseudoexfoliation.

a Twenty-nine subjects are excluded; two subjects were missing their right eyes.

The stratified analyses are presented in Table 5. OAG (OR_MH_ 8.97; 95% CI 3.84–20.9), PEX (OR_MH_ 2.40; 95% 1.53–3.76), and diabetes (OR_MH_ 1.83; 95% CI 1.08–3.09) were related to an IOP ≥20 mm Hg, while age, sex, cataract, smoking, systemic hypertension, and ischemic heart disease were not. The factors in Table 5 were tested in logistic regression models. The results of a model including age, sex, OAG, PEX, smoking status, and diabetes were almost identical to that of the stratified analyses (data not shown). There was no indication of interaction in the models.

**Table 5:** Odds ratios for intraocular pressure ≥20 mm Hg in either eye in 733 participants in the Tierp Glaucoma Survey, adjusted for age and sex.^a^

Characteristics		No. of cases (*n* = 129)	OR_M-H_	95% CI
Age ≥70 years^b^	No	76	1.00	
	Yes	53	0.75	0.51–1.10
Male sex^c^	No	72	1.00	
	Yes	57	0.83	0.57–1.22
Open-angle glaucoma, either eye^b^	No	114	1.00	
	Yes	15	8.97	3.84–20.93
Pseudoexfoliation, either eye	No	93	1.00	
	Yes	36	2.40	1.53–3.76
Cataract, either eye	No	90	1.00	
	Yes	39	1.04	0.68–1.59
Smoking status	Never smoked	78	1.00	
	Past smoker	28	1.61	0.93–2.78
	Current smoker	23	1.43	0.82–2.49
Diabetes	No	106	1.00	
	Yes	23	1.83	1.08–3.09
Hypertension, treated	No	90	1.00	
	Yes	39	1.16	0.76–1.77
Ischaemic heart disease	No	107	1.00	
	Yes	22	1.32	0.79–2.22

CI: confidence interval; OR_M-H_: Mantel-Haenszel adjusted odds ratio.

a Twenty-seven subjects are excluded from the analyses;

b Adjusted for sex;

c Adjusted for age.

## Discussion

In this study, the distribution of IOP was close to that of other European-derived populations of the same age (6, 7). Likewise, in agreement with other population surveys (10–12), PEX was associated with increased IOP, defined as a pressure ≥20 mm Hg in either eye. Thus, the high prevalence of PEX in the examined population (16%) had no significant impact on the distribution of IOP.

To the best of our knowledge, the current study, including a defined population, was the first to explore the effect of which eye is measured first. Although the difference was small, in subjects 65–69 years of age, the mean pressure was higher in eyes randomly assigned to be measured first. Furthermore, analysis of variance revealed a significant interaction between measuring the right eye first and the left eye second. It is well known that repeated applanation tonometry reduces the pressure (19–22). However, the reason for the pressure decreasing is not fully understood. One explanation presupposes a passing stage of initial tension in subjects being examined (23). Interestingly, psychological stress has been proven to result in an increase of the IOP in healthy individuals (24). The results of the present study support the idea of stress as the cause of this phenomenon. However, even if the findings present new knowledge, they do not have any apparent clinical implication other than a recommendation to repeat the measurements if there is a noteworthy difference in the IOP between the two eyes.

Increased IOP has frequently been related to OAG in population surveys (15, 25–27). In fact, a strong association was demonstrated also in the current study, where an IOP ≥20 mm Hg increased the risk of having OAG 9-fold (Table 5). Moreover, in accordance with the studies in Framingham and Beaver Dam (6, 7), we did not find any relationship between age or sex and the distribution of IOP.

Systemic hypertension has consistently been associated with an increased IOP in many studies (7, 8, 28, 29). This was not the case in the present study, in which only individuals treated for hypertension based on the medical records were classified as exposed. In contrast, the blood pressure was measured in the other population studies referred to above. It is impossible to speculate on what effect the different methods might have had on the estimates. Lack of statistical power may also have affected the study in Tierp. For this reason, the results should be interpreted with some caution.

At present, a positive relationship between diabetes mellitus and IOP is well established in the literature (29–32). In larger population surveys, diabetes was usually diagnosed either from a self-reported history of taking medication or the determination of plasma glucose levels in blood samples. In this study, an IOP ≥20 mm Hg increased the risk of having been diagnosed with diabetes by 83%. Why subjects with diabetes have a higher IOP is unclear. An explanation often mentioned implies that raised glucose levels induce an osmotic gradient, attracting fluid into the intraocular space, resulting in increased pressure (30). A genetic link between the two disorders has also been suggested (33).

Our study has several strengths, including its community-based design, high participation rate, and the use of a detailed protocol. The eye pressures were taken by an experienced assistant, and all eye examinations conducted by the same glaucoma specialist, who was masked to the result of the pressure readings and the visual field testing. Furthermore, a visual field defect was required for a diagnosis of OAG. Nevertheless, as with many epidemiologic studies, the research was limited in several respects.

Most importantly, compared with many other population studies, the Tierp Glaucoma Survey was a small study, limiting its statistical power to provide reliable estimates on some of the predictors of increased IOP. However, the IOP measurements delivered sufficient data for an accurate description of the IOP distribution in the examined population, which was the main issue of this study. Furthermore, the study only involved individuals aged 65–74-years-old. Nonetheless, there are no reports on significant age differences in the distribution of IOP in other European-derived population.

There is always a risk of misclassification of exposure in cross-sectional studies when data are based on self-reports, which was the case regarding smoking habits. This type of information bias should be non-differential, thereby ‘diluting’ the relationship between increased IOP and possible predictors in the analyses. The lack of association with systemic hypertension in this study was possibly an effect of non-differential misclassification.

In conclusion, in this population-based study on individuals aged 65–74-years-old in Sweden, the distribution of IOP was close to that of the Framingham and Beaver Dam studies. The pressure in the first measured eye was higher than the pressure in the second eye. Increased IOP was strongly related to untreated OAG and PEX.
